# Multiple functions of DDX3 RNA helicase in gene regulation, tumorigenesis, and viral infection

**DOI:** 10.3389/fgene.2014.00423

**Published:** 2014-12-05

**Authors:** Yasuo Ariumi

**Affiliations:** Ariumi Project Laboratory, Center for AIDS Research – International Research Center for Medical Sciences, Kumamoto UniversityKumamoto, Japan

**Keywords:** DDX3, HCV, HIV-1, innate immunity, RNA helicases, stress granules, translation, tumor suppressor

## Abstract

The DEAD-box RNA helicase DDX3 is a multifunctional protein involved in all aspects of RNA metabolism, including transcription, splicing, mRNA nuclear export, translation, RNA decay and ribosome biogenesis. In addition, DDX3 is also implicated in cell cycle regulation, apoptosis, Wnt-β-catenin signaling, tumorigenesis, and viral infection. Notably, recent studies suggest that DDX3 is a component of anti-viral innate immune signaling pathways. Indeed, DDX3 contributes to enhance the induction of anti-viral mediators, interferon (IFN) regulatory factor 3 and type I IFN. However, DDX3 seems to be an important target for several viruses, such as human immunodeficiency virus type 1 (HIV-1), hepatitis C virus (HCV), hepatitis B virus (HBV), and poxvirus. DDX3 interacts with HIV-1 Rev or HCV Core protein and modulates its function. At least, DDX3 is required for both HIV-1 and HCV replication. Therefore, DDX3 could be a novel therapeutic target for the development of drug against HIV-1 and HCV.

## INTRODUCTION

DDX3 belongs to the DEAD (D-E-A-D: Asp-Glu-Ala-Asp)-box RNA helicase family, which is an ATPase-dependent RNA helicase, is found in various organisms from yeast to human (Cordin et al., 2006; Linder and Lasko, 2006; Linder, 2008; Jankowsky, 2011). DDX3 has two homologs designated DDX3X (DBX) and DDX3Y (DBY), which were located on X and Y chromosomes, respectively (Lahn and Page, 1997; Park et al., 1998; Kim et al., 2001). DDX3X is ubiquitously expressed in most tissues, while the expression of DDX3Y protein is limited to the male germline (Ditton et al., 2004) and DDX3Y seems to be involved in male fertility (Leory et al., 1989; Mazeyrat et al., 1998; Foresta et al., 2000). DDX3 is involved in various RNA metabolism, including transcription, translation, RNA splicing, RNA transport, and RNA degradation (Chang and Liu, 2010; Schröder, 2010).

## REGULATION OF GENE EXPRESSION BY DDX3

DDX3 regulates gene expression at different levels, such as transcription, splicing, mRNA export, and initiation of translation. First, DDX3 participates in transcriptional regulation of gene promoters. Indeed, DDX3 up-regulates the interferon (IFN) β promoter (Soulat et al., 2008) and the p21^waf1/cip1^ promoter (Chao et al., 2006), respectively. DDX3 binds to the transcription factor Sp1 and enhance the p21^waf1/cip1^ promoter. On the other hand, DDX3 down-regulates the E-cadherin promoter (Botlagunta et al., 2008). *In vivo* association of DDX3 with the E-cadherin or the IFNβ promoter was demonstrated by chromatin immunoprecipitation assay. Second, DDX3 seems to contribute to splicing. DDX3 associates with spliced mRNAs in an exon junction complex (EJC)-dependent manner (Merz et al., 2007) and DDX3 contains C-terminal RS-like domain, which is stretches of protein sequence rich in arginine and serine residues and is found in splicing factors. Third, DDX3 contributes to the nuclear export of RNA. DDX3 shuttles between the cytoplasm and the nucleus (Owsianka and Patel, 1999; Yedavalli et al., 2004; Lai et al., 2008; Schröder et al., 2008). Accordingly, DDX3 interacts with two nuclear export shuttle protein: CRM1 as a receptor for protein containing the nuclear export signal (NES) and tip-associated protein (TAP) as the major receptor for mRNA export (Yedavalli et al., 2004; Lai et al., 2008). DDX3 interacts with CRM1 and functions in the human immunodeficiency virus type 1 (HIV-1) Rev-dependent nuclear export of HIV-1 mRNA (Yedavalli et al., 2004). Depletion of TAP resulted in nuclear accumulation of DDX3, suggesting DDX3 exports along with messenger ribonucleoprotein (mRNP) to the cytoplasm via the TAP-mediated pathway (Lai et al., 2008).

Forth, DDX3 plays a role in translational regulation. DDX3 localizes in cytoplasmic stress granules under stress conditions (Lai et al., 2008; Shih et al., 2012), suggesting a role for DDX3 in translational control. DDX3 represses the cap-dependent translation by trapping eIF4E in a translationally inactive complex to block an interaction with eIF4G (Shih et al., 2008), indicating that DDX3 acts as a translational suppressor. Since depletion of DDX3 does not significantly affect general translation, DDX3 may be dispensable for general mRNA translation (Lai et al., 2008). Indeed, DDX3 associates with eIF4E together with several translation initiation factors, including eIF4a, eIF4G, eIF2a, eIF3, and poly(A)-binding protein (PABP), and facilitates translation of mRNA containing structured 5′ untranslated region (UTR; Lai et al., 2008; Shih et al., 2012; Soto-Rifo et al., 2012). In contrast, others reported that primary function for DDX3 is in protein translation via an interaction with eIF3 (Lee et al., 2008). Accordingly, DDX3 interacts with eIF3 and 40S ribosome to support the assembly of functional 80S ribosome (Geissler et al., 2012). The yeast DDX3 homolog, Ded1, also modulates translation by the formation of a translation initiation factor eIF4F-mRNA complex (Hilliker et al., 2011). Taken together, DDX3 modulates the protein translation.

Finally, DDX3 interacts with Ago2, which is an essential factor in RNA interference (RNAi) pathway that cleaves target mRNA, and acts as an essential factor involved in RNAi pathway (Kasim et al., 2013).

## DDX3 IN CELL CYCLE REGULATION AND TUMORIGENESIS

It has been indicated a role of DDX3 in cell cycle regulation, apoptosis, and tumorigenesis. In the temperature-sensitive DDX3 mutant hamster cell line tsET24 or the DDX3 knockdown cells, cell cycle was impedes transition from G_1_ to S-phase (Fukumura et al., 2003; Lai et al., 2010). DDX3 enhances cyclin E1 during cell cycle by a translational regulation (Lai et al., 2010). On the other hand, DDX3 regulates the cell cycle by inhibiting cyclin D1 and causing cell cycle arrest (Chao et al., 2006). DDX3 is known to be phosphorylated by cyclin B/cdc2 at threonine 204 to inhibit the function (Sekiguchi et al., 2007). Furthermore, DDX3 interacts with DDX5, which colocalizes with it in the cytoplasm through the phosphorylation of both proteins during G_2_/M phase of cell cycle (Choi and Lee, 2012), indicating the cell cycle-dependent regulation of DDX3 localization and the function. During mouse early embryonic development, DDX3 also regulates cell survival and cell cycle (Li et al., 2014b).

It has been indicated the oncogenic role of DDX3 in breast cancer (Botlagunta et al., 2008). Activation of DDX3 by benzo[a]pyrene diol epoxide (BPDE) present in tobacco smoke, can promote growth, proliferation and neoplastic transformation of breast epithelial cells. Consistent with this finding, overexpression of DDX3 induced an epithelial-mesenchymal-like transformation, exhibited increased motility and invasive properties, and formed colonies in soft agar assays. In addition, DDX3 is recruited to the E-cadherin promoter and represses the E-cadherin expression resulting the increased cell migration and metastasis (Botlagunta et al., 2008). Similarly, DDX3 also modulates cell adhesion, motility and cancer cell metastasis via Rac1-mediated signaling pathway (Chen et al., 2014). In fact, DDX3 knockdown reduces the cell migration, the invasive and metastatic activities, suggesting that DDX3 is required for metastasis and the oncogenic role of DDX3 in malignant cancers. The DDX3 knockdown also reduces the expression of levels of both Rac1 and β-catenin. DDX3 regulates Rac1 mRNA translation through an interaction with its 5′UTR and affects β-catenin protein stability in Rac1-dependent manner. In response to Wnt signaling, DDX3 binds to casein kinase (CK) 1ε and stimulates CK1ε-mediated phosphorylation of the Wnt effector disheveled and thereby activates β-catenin (Cruciat et al., 2013), indicating a role of DDX3 as a regulator of Wnt-β-catenin network. Moreover, DDX3 may aid cancer progression by promoting increased levels of the transcription factor Snail (Sun et al., 2011). Snail is known to repress the expression of cellular adhesion proteins, leading to increased cell migration and metastasis of many types of cancer. In addition, recent study reported that positive DDX3 expression is significantly associated with large tumor size and high TNM (Tumor, Node, and Metastasis) stage, invasion, lymph node metastasis in gallbladder cancers (Miao et al., 2013), suggesting that DDX3 is a biomarker for metastasis and poor prognosis of gallbladder cancers. TNM classification is an anatomically based staging system that records the primary and regional nodal extent of the tumor and the absence or presence of metastases.

Hypoxia is a major characteristic of solid tumors and affects gene expression, which greatly impacts cellular and tumor tissue physiology particularly respiration and metabolism. Expression of hypoxia-responsive genes is predominantly regulated by hypoxia inducible factors (HIFs). DDX3 is aberrantly expressed in breast cancer cells ranging from weakly invasive to aggressive phenotypes (Botlagunta et al., 2011). HIF-1 binds to the DDX3 promoter and enhances the DDX3 expression (Botlagunta et al., 2011), indicating a DDX3 as a hypoxia inducible gene.

In contrast, DDX3 has been proposed to be a tumor suppressor (McGivern and Lemon, 2009). In fact, DDX3 inhibits colony formation in various cell lines and down-regulates cyclin D1 and up-regulates the p21^waf1/cip1^ promoter (Chao et al., 2006). DDX3 expression is deregulated in hepatocellular carcinoma (HCC; Chang et al., 2006; Chao et al., 2006). Loss of DDX3 leads to enhanced cell proliferation and reduced apoptosis (Chang et al., 2006). Similarly, loss of DDX3 by p53 inactivation promotes tumor malignancy via the MDM2/Slug/E-cadherin pathway and consequently results in poor patient outcome in non-small-cell lung cancer (Wu et al., 2014). In addition, DDX3 contributes to both antiapoptotic and proapoptotic actions. Death receptors are found to be capped by an antiapoptotic protein complex containing GSK3, DDX3 and cIAP-1 and DDX3 protects from apoptotic signaling (Sun et al., 2008). In contrast, DDX3 also associates with p53, increases p53 accumulation, and positively regulates DNA damage-induced apoptosis (Sun et al., 2013). Furthermore, reduced p21^waf1/cip1^ via alteration of p53-DDX3 pathway is associated with poor relapse-free survival in early stage human papillomavirus-associated lung cancer (Wu et al., 2011). Thus, p21^waf1/cip1^ is considered to act as a tumor suppressor. Since low/negative DDX3 expression in tumor cells is significantly associated with aggressive clinical manifestations, low/negative expression of DDX3 might predict poor prognosis in oral cancer patients (Lee et al., 2014).

Altogether, DDX3 has both tumor suppression and oncogenic properties. This may reflect on the cell type used in their experiments. Further studies are necessary to clarify the potential role of DDX3 in cell growth regulation. These studies may shed a light on the development of drugs for chemotherapy against cancer and viral infection described below.

## DDX3 AS A TARGET OF VIRUSES

DDX3 has been implicated in a target of several viruses, including hepatitis C virus (HCV), HIV-1, hepatitis B virus (HBV), West Nile virus (WNV), Japanese encephalitis virus, norovirus, pestivirus, vaccinia virus, and cytomegalovirus (**Table 1**). DDX3 is required for several RNA viral replication such as HCV and HIV-1, while DDX3 restricts HBV replication. At least, DDX3 may be a therapeutic target for anti-viral drug against HCV and HIV-1.

**Table 1:** DDX3 as a target of viruses.

Virus	Effect of DDX3 on viral replication	Viral binding protein	Cellular function
HCV	Up-regulation	Core	Translational regulation
HIV-1	Up-regulation	Rev Tat	Nuclear export of mRNA Translational regulation
HBV	Down-regulation	Pol	Inhibition of IFN induction
Vaccinia virus	?	K7	Inhibition of IFN induction
WNV	Up-regulation	?	?

*DDX3 interacts with several RNA virus including hepatitis C virus (HCV), human immunodeficiency virus type 1 (HIV-1), hepatitis B virus (HBV), vaccinia virus, and West Nile virus (WNV). DDX3 is required for HCV, HIV-1, WNV replication, while DDX3 restricts HBV replication. Furthermore, these viral proteins suppress the DDX3-mediated type I IFN induction though an interaction with DDX3.*

## REQUIREMENT OF DDX3 IN HCV LIFE CYCLE

Hepatitis C virus is a causative agent of chronic hepatitis, which progresses to liver cirrhosis and HCC. HCV is an enveloped virus with a positive single-stranded 9.6 kb RNA genome, which encodes a large polyprotein precursor of ∼3,000 amino acid residues (Kato et al., 1990). This polyprotein is cleaved by a combination of the host and viral proteases into at least 10 proteins in the following order: core, envelope 1 (E1), E2, p7, non-structural 2 (NS2), NS3, NS4A, NS4B, NS5A, and NS5B (Hijikata et al., 1991, 1993). The HCV core protein is a viral structural protein, which forms the viral nucleocapsid, is targeted to lipid droplets (LDs). Recently, LDs have been found to be an important cytoplasmic organelle for HCV production (Miyanari et al., 2007). Budding is an essential step in the life cycle of enveloped viruses. HCV utilizes the endosomal sorting complex required for transport (ESCRT) system as the budding machinery (Ariumi et al., 2011b).

**FIGURE 1:**
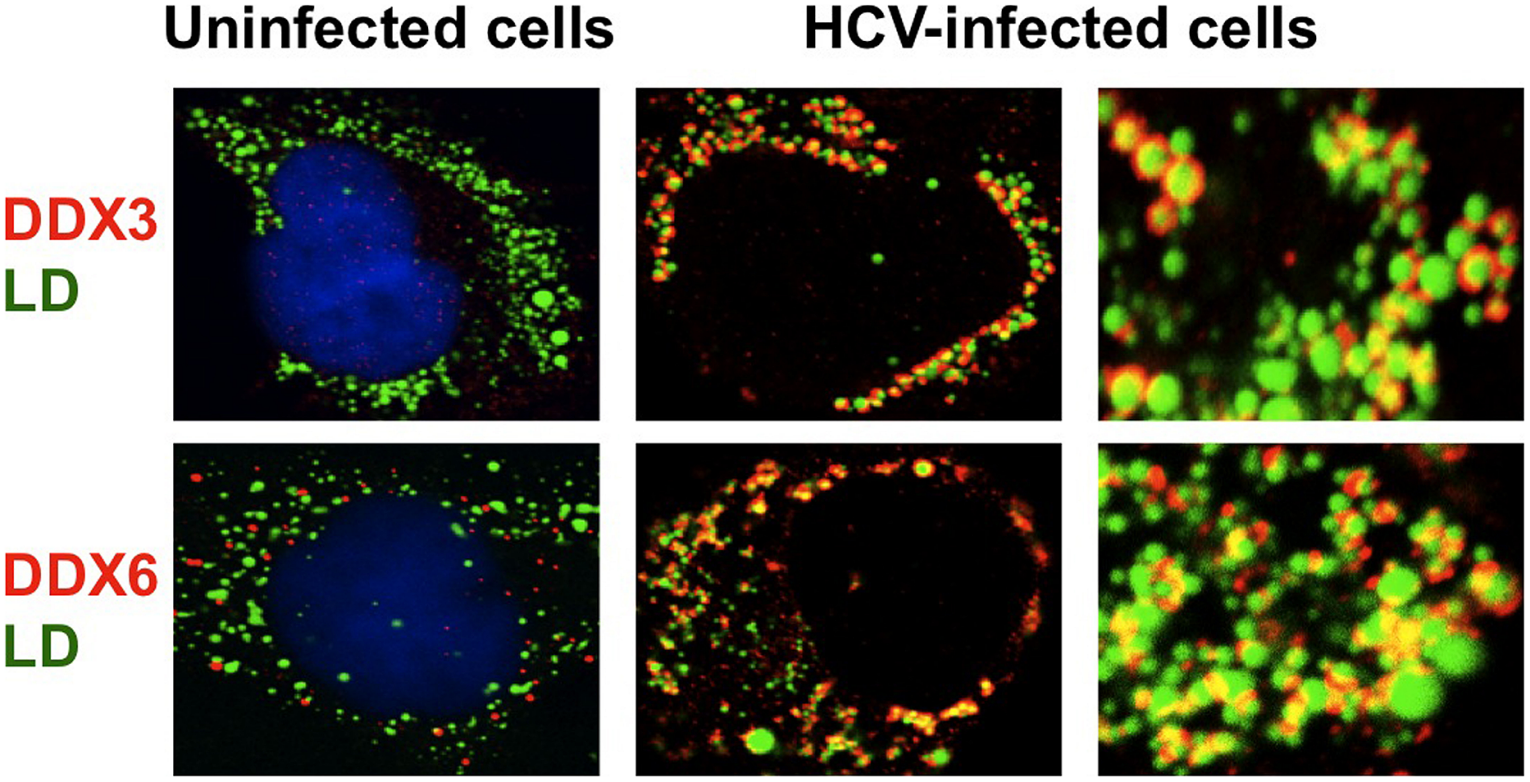
**Dynamic recruitment of DDX3 and DDX6 around lipid droplets (LDs) in response to HCV-JFH1 infection.** Cells were fixed 60 h post-infection with HCV (JFH1 strain) and stained with either anti-DDX3 or anti-DDX6 antibody and were then visualized with Cy3 (red). Lipid droplets were specifically stained with fluorescent lipophilic dye BODIPY 493/503 (green; Listenberger and Brown, 2007) and nuclei were stained with DAPI (blue), respectively. Images were visualized using confocal laser scanning microscopy. The two-color overlay images are also exhibited (merged). Colocalization is shown in yellow. High magnification image is also shown.

Several DEAD-box RNA helicases have been shown to interact with HCV proteins and regulate the HCV replication (Schröder, 2010; Upadya et al., 2014). DDX3 was identified as an HCV core-binding protein by yeast two-hybrid screening (Mamiya and Worman, 1999; Owsianka and Patel, 1999; You et al., 1999). HCV core protein was the first viral protein to be described as a DDX3-binding protein. HCV core binds to the C-terminal RS-like domain of DDX3 and the interaction is mediated by the N-terminal 59 amino acid residues of HCV core. DDX3 and HCV core colocalized in distinct spots in the perinuclear region of the cytoplasm. However, these studies lack evidence regarding the functional relevance of the DDX3-HCV core interaction in HCV replication and the HCV-associated liver diseases. Recent studies have demonstrated that DDX3 is required for HCV replication (Ariumi et al., 2007; Randall et al., 2007). The accumulation of both genome-length HCV RNA (HCV-O strain, genotype 1b; Ikeda et al., 2005) and its replicon RNA were significantly suppressed in the DDX3 knockdown cells. As well, HCV infection (JFH1 strain, genotype 2a; Wakita et al., 2005) was also suppressed in the DDX3 knockdown cells. Notably, HCV infection dynamically redistributes DDX3 to the HCV production site around LDs and colocalizes with HCV core (**Figure 1**; Ariumi et al., 2011a). However, the specific interactions between DDX3 and HCV core and the functional importance of these interactions for the HCV viral life cycle remain unclear. In this regard, Mutagenesis studies located a single amino acid in the N-terminal domain of JFH1 core that when changed to alanine significantly abrogated this interaction. Surprisingly, this mutation did not alter infectious virus production and RNA replication, indicating that the core-DDX3 interaction is dispensable in the HCV life cycle (Angus et al., 2010). On the other hand, there is a contradictory report that the inhibition of HCV replication due to expression of the green fluorescent protein (GFP) fusion to HCV core protein residues 16–36 can be reversed by overexpression of DDX3 (Sun et al., 2010). These results suggest that the protein interface on DDX3 that binds the HCV core protein is important for replicon maintenance. However, infection of HuH-7 cells by HCV (JFH1) was not affected by expression of the GFP fusion protein. These results suggest that the role of DDX3 in HCV infection involves aspects of the viral life cycle that vary in importance between HCV genotypes. Therefore, the exact contribution of HCV core-DDX3 interaction remains to be determined.

In addition to DDX3, other DEAD-box RNA helicases DDX1, DDX5, and DDX6 have been involved in the HCV life cycle (Goh et al., 2004; Tingting et al., 2006; Jangra et al., 2010; Ariumi et al., 2011a; Kuroki et al., 2013). DDX1 bound to both the HCV 3′UTR and the HCV 5′UTR and DDX1 knockdown caused a marked reduction in the replication of subgenomic replicon RNA (Tingting et al., 2006). Furthermore, DDX5 was identified as an HCV NS5B RNA-dependent RNA polymerase-binding protein by yeast two-hybrid screening (Goh et al., 2004). Depletion of endogenous DDX5 correlated with a reduction in the transcription of negative strand HCV RNA, suggesting that DDX5 participates in the HCV RNA replication. Overexpression of HCV NS5B or the HCV infection redistributes DDX5 from the nucleus to the cytoplasm. Moreover, recent study reported that knockdown of DDX5 reduces HCV (JFH1) virus production in the supernatant, suggesting that DDX5 is important for a late stage of the HCV life cycle (Kuroki et al., 2013).

**FIGURE 2:**
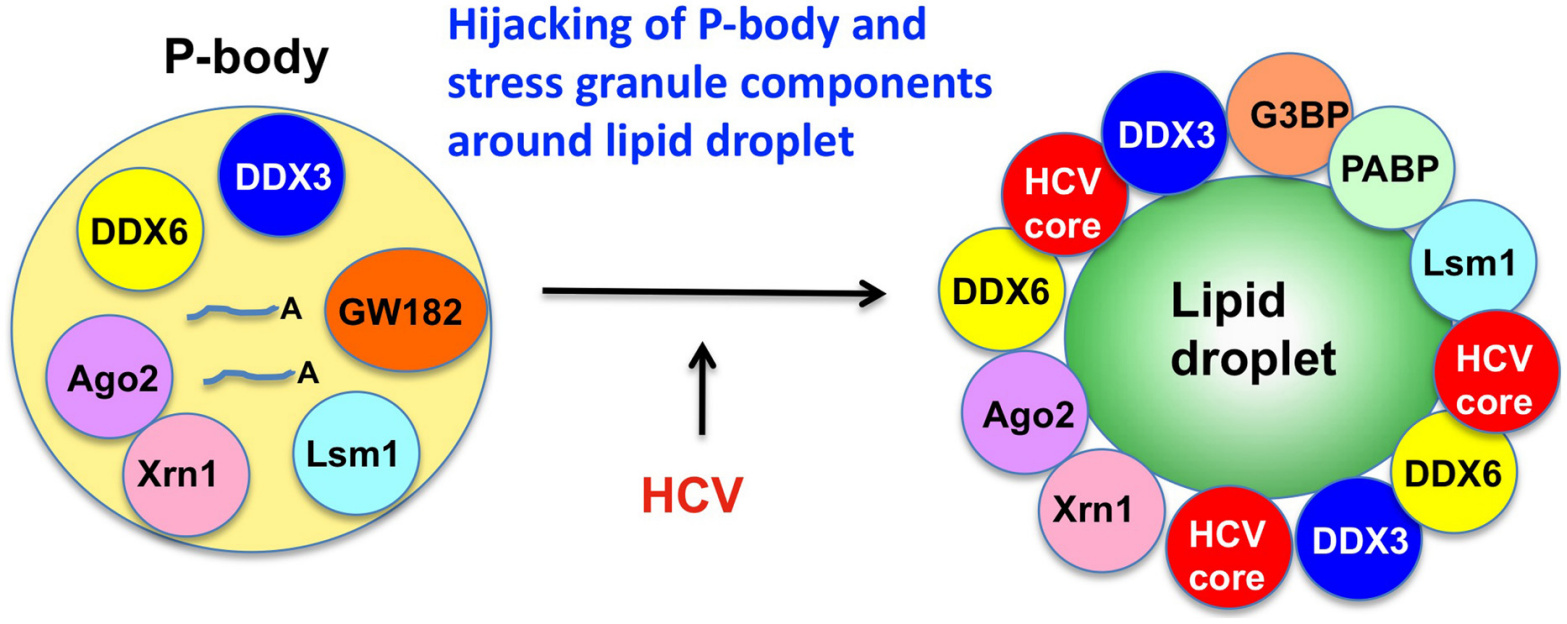
**Hijacking of P-body components around LD by HCV.** HCV disrupts the P-body and hijacks the P-body components including DDX3, DDX6, Ago2, Xrn1, and Lsm1 around LD, an HCV production site.

The microRNA miR122 and DDX6/Rck/p54, a microRNA effector, have been implicated in HCV replication (Jopling et al., 2005; Scheller et al., 2009; Jangra et al., 2010; Ariumi et al., 2011a). The liver-specific and abundant miR-122 interacts with the 5′UTR of the HCV RNA genome and facilitates the HCV replication (Jopling et al., 2005). DDX6 interacts with the eukaryotic initiation factor 4E (eIF-4E) to repress the translational activity of mRNP. Furthermore, DDX6 regulates the activity of the decapping enzymes DCP1 and DCP2 and interacts directly with Argonaute-1 (Ago1) and Ago2 in the microRNA-induced silencing complex (miRISC) and is involved in RNA silencing. DDX6 predominantly localizes in the discrete cytoplasmic foci termed processing (P)-body. Thus, the P-body may play a role in the translation repression and mRNA decay machinery (Parker and Sheth, 2007; Beckham and Parker, 2008). The knockdown of DDX6 was found to reduce the accumulation of intracellular HCV RNA and infectious HCV production, indicating that DDX6 is essential for the HCV RNA replication (Scheller et al., 2009; Jangra et al., 2010; Ariumi et al., 2011a). Notably, HCV (JFH1) infection disrupts the P-body formation of DDX3, DDX6, Lsm1, Xrn1, PATL1, and Ago2 and dynamically redistributes them to the HCV production site around LDs (**Figure 2**; Ariumi et al., 2011a), indicating that HCV hijacks the P-body components around LDs and regulates the HCV replication and translation. Recent studies suggested that DDX3 is also required for WNV, Japanese encephalitis virus, norovirus, and pestivirus (Vashist et al., 2012; Chahar et al., 2013; Jefferson et al., 2014; Li et al., 2014a; Tsai and Lloyd, 2014). Similarly, P-body components LSM1, GW182, DDX3, DDX6, and XRN1 are also recruited to WNV replication sites and positively regulate viral replication (Chahar et al., 2013).

On the other hand, recent studies have suggested a potential role of DDX3 and DDX5 in the pathogenesis of HCV-related liver diseases. DDX3 expression is deregulated in HCC (Chang et al., 2006; Chao et al., 2006) and single-nucleotide polymorphisms were identified in the DDX5 genes that were associated with an increased risk of advanced fibrosis in patients with chronic hepatitis C (Huang et al., 2006). DDX3 has been proposed to be a tumor suppressor (McGivern and Lemon, 2009). In fact, DDX3 inhibits colony formation in various cell lines, including human hepatoma HuH-7, and up-regulates the p21^waf1/cip1^ promoter (Chao et al., 2006). Therefore, HCV core protein might overcome the DDX3-mediated cell growth arrest and down-regulate p21^waf1/cip1^ through an interaction with DDX3, and it might be involved in the development of HCC.

## DDX3 IS ESSENTIAL FOR HIV-1 REPLICATION

Human immunodeficiency virus type 1 is the causative agent of acquired immune deficiency syndrome (AIDS). HIV-1 is a retrovirus with a positive strand RNA genome of 9 kb which encodes nine polypeptides, structural proteins, Gag (group specific antigen), Pol (polymerase) and Env (envelope), the accessory proteins, Vif, Vpu, Vpr, and Nef, and the regulatory proteins, Tat and Rev. The gene expression of HIV-1 is regulated transcriptionally by Tat through its binding to a nascent viral *trans*-activation responsive (TAR) RNA (Berkhout et al., 1989; Jeang et al., 1999), and post-transcriptionally by Rev through its association with Rev-responsive element (RRE) in the *env* gene (Hope and Pomerantz, 1995; Pollard and Malim, 1998; Cullen, 2003). Since the intron-containing host RNA cannot leave the nucleus before it is completely spliced, HIV-1 needs to evade host surveillance system to export unspliced or partially spliced viral RNA into cytoplasm and produce HIV-1 structural proteins and accessory proteins. For this, Rev contains a leucine-rich NES that recruits nuclear export receptor CRM1 (Hope and Pomerantz, 1995; Pollard and Malim, 1998; Cullen, 2003). Upon binding to the RRE together with the GTP-bound form of Ran (Ran-GTP), CRM1 forms the nuclear export complex and Rev-CRM1-RRE-Ran-GTP complex exports unspliced or partially spliced HIV-1 RNA from the nucleus to the cytoplasm.

Several viruses are known to carry their own RNA helicases to facilitate the replication of their viral genome, including HCV, flavivirus, severe acute respiratory syndrome (SARS) coronavirus, rubella virus, and alphavirus, however, HIV-1 does not encode own RNA helicase (Utama et al., 2000; Kwong et al., 2005). Thus, host RNA helicases may be involved in HIV-1 replication at multiple stages, including the reverse transcription of HIV-1 RNA, HIV-1 mRNA transcription, the nucleus-to-cytoplasm transport of HIV-1 mRNA, and HIV-1 RNA packaging (Cochrane et al., 2006; Lorgeoux et al., 2012).

**FIGURE 3:**
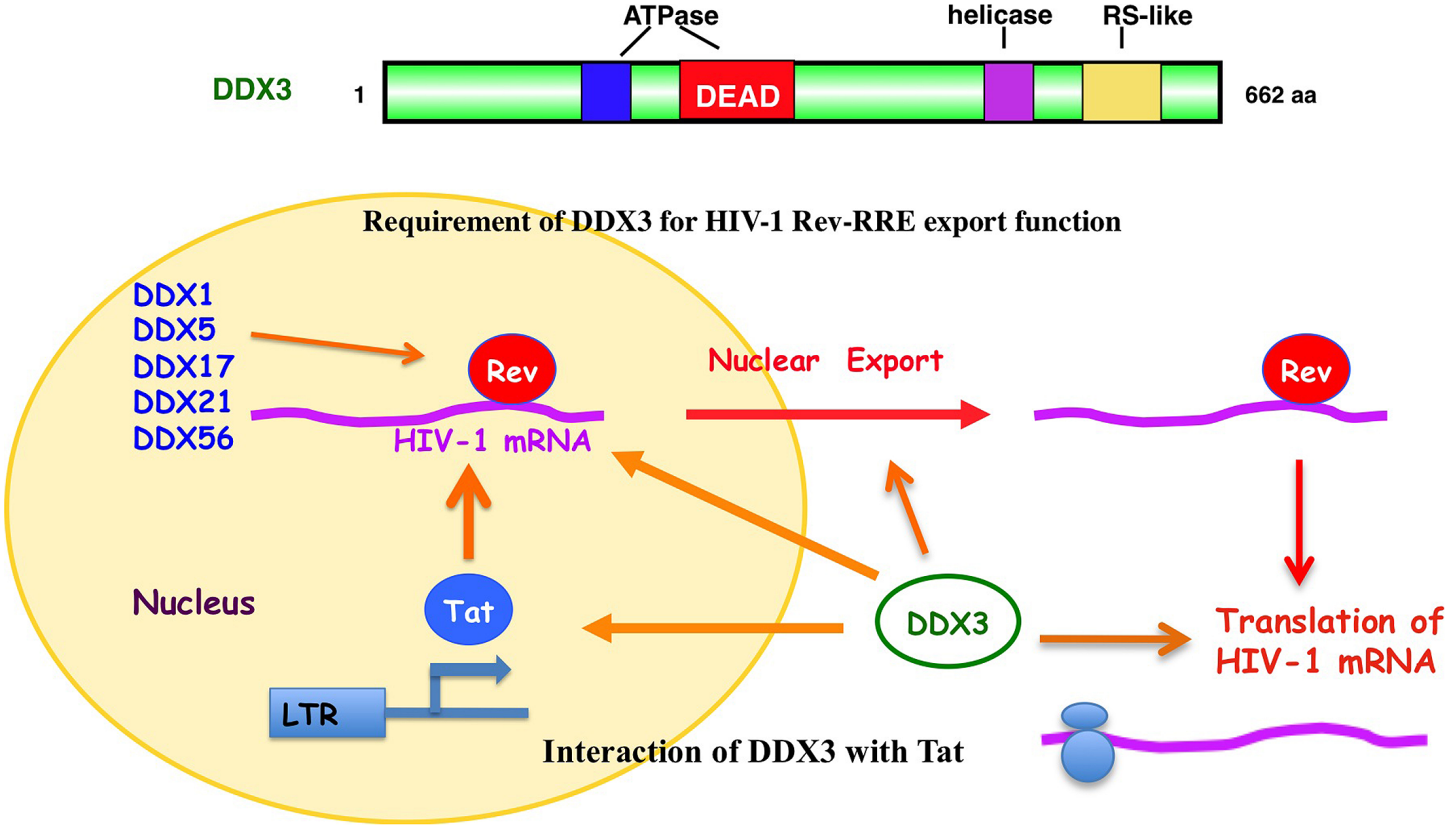
**Role of DDX3 in the HIV-1 gene expression.** DDX3 interacts with HIV-1 Rev and facilitates the Rev-dependent nuclear export of HIV-1 mRNA. DDX3 interacts with Tat and contributes to the translation of HIV-1 mRNA. Other DEAD-box RNA helicases, including DDX1, DDX5, DDX17, DDX21, and DDX56, also interact with HIV-1 Rev and facilitate its function.

In fact, DDX3 was first found to involve in the Rev-dependent nuclear export of unspliced and partially spliced HIV-1 RNAs (**Figure 3**; Yedavalli et al., 2004). Over-expression of DDX3 enhanced the Rev-dependent nuclear export function. Conversely, knockdown of DDX3 or expression of dominant negative mutant of DDX3 significantly suppressed the Rev function as well as HIV-1 replication (Yedavalli et al., 2004; Ishaq et al., 2008). Rev is co-immunoprecipitated with DDX3. DDX3 is a nucleo-cytoplasmic shuttling protein, which binds CRM1 and localizes to nuclear membrane pores.

In addition to DDX3, another DEAD-box RNA helicase DDX1 also associates with Rev and promotes the Rev-dependent RNA nuclear export function (Fang et al., 2004). DDX1 interacts with Rev via the N-terminal domain, suggesting a role of DDX1 in initial complex assembly. DDX1 promotes Rev oligomerization on the RRE through this interaction (Robertson-Anderson et al., 2011). Thus, DDX1 and DDX3 act sequentially in the Rev-dependent RNA nuclear export. DDX1 first binds to Rev and promotes Rev oligomerization on the RRE. Then, the oligomerized Rev recruits the CRM1/DDX3 complex that subsequently exports the RRE-containing HIV-1 RNAs into the cytoplasm (Lorgeoux et al., 2012). In addition to DDX1 and DDX3, we and other group recently reported that other RNA helicases, including DDX5, DDX17, DDX21, DHX36, DDX47, DDX56, and RNA helicase A (RHA) associate with the Rev-dependent nuclear export function (**Figure 3**; Li et al., 1999; Naji et al., 2012; Yasuda-Inoue et al., 2013a; Zhou et al., 2013). Furthermore, DDX3 interacts with DDX5 and synergistically enhances the Rev-dependent nuclear export. As well, combination of other distinct DDX RNA helicases such as DDX1 and DDX3 also synergistically facilitates the Rev function (Yasuda-Inoue et al., 2013a) suggesting that a set of distinct Rev-interacting DEAD-box RNA helicases cooperate to modulate the HIV-1 Rev function.

On the other hand, HIV-1 Tat activates the HIV-1 RNA synthesis. Tat binds to the TAR RNA and recruits several host factors including p300/CREB-binding protein (p300/CBP), p300/CBP-associated factor (PCAF), SWI/SNF chromatin-remodeling complex, and positive transcription elongation factor b (P-TEFb) to stimulate both transcription initiation and elongation (Jeang et al., 1999; Ariumi et al., 2006; Lorgeoux et al., 2012). P-TEFb contains cyclin T1 and cyclin-dependent kinase 9 (CDK9). CDK9 hyperphosphorylates the C-terminal domain (CTD) of RNA Pol II and activates transcription elongation. The Werner syndrome (WRN) helicase and RHA were reported to act as co-factors of Tat and enhance the HIV-1 gene expression (Fujii et al., 2001; Sharma et al., 2007). In addition to WRN and RHA, DDX3 interacts with Tat (**Figure 3**; Lai et al., 2013; Yasuda-Inoue et al., 2013b). Tat is partially targeted to cytoplasmic stress granules upon DDX3 overexpression or cell stress conditions, suggesting a potential role of Tat/DDX3 complex in translation. Accordingly, Tat remains associated with translating mRNAs and facilitates translation of mRNAs containing the HIV-1 5′UTR. In this regard, DDX3 is essential for translation of HIV-1 genomic RNA (gRNA; **Figure 3**; Soto-Rifo et al., 2012). DDX3 directly binds to the HIV-1 5′UTR and interacts with eIF4G and PABP but lacking the major cap-binding proteins eIF4E in large cytoplasmic RNA granules (Soto-Rifo et al., 2013), indicating that DDX3 promotes the HIV-1 gRNA translation initiation in an eIF4E-independent manner.

Both HIV-1 and HCV have been shown to utilize DDX3 as a cofactor for viral genome replication. Therefore, DDX3 could be an important therapeutic target for development of anti-viral drug (Kwong et al., 2005). Indeed, small molecule inhibitors were used to inhibit ATPase activity of DDX3 with anti-HIV-1 activity (Maga et al., 2008, 2011; Yedavalli et al., 2008; Radi et al., 2012).

## DDX3 RESTRICTS HBV REPLICATION

Hepatitis B virus is also the causative agent of chronic hepatitis, which progresses to liver cirrhosis and HCC worldwide. HBV belongs to hepadnavirus family and contains a small partially double-stranded circular DNA genome of 3.2 kb. Even though HBV is a DNA virus, HBV replicates its DNA genome via reverse transcription. Upon HBV infection, the HBV DNA is converted into covalently closed circular DNA (cccDNA) as the template for the viral transcription. Pregenomic RNA (pgRNA) of 3.5 kb is selectively packaged into nucleocapsid together with HBV Pol. The pgRNA is reverse transcribed by HBV Pol to generate relaxed circular (RC) DNA. The HBV reverse transcription occurs entirely within nucleocapsid following encapsidation.

Recently, it was shown that DDX3 specifically binds to the HBV Pol and is incorporated into nucleocapsid together with HBV Pol (Wang et al., 2009). However, unlike HIV-1 and HCV replication, which is enhanced by DDX3 (Yedavalli et al., 2004; Ariumi et al., 2007; Randall et al., 2007), HBV reverse transcription was inhibited by DDX3. In addition, recent study reported that DDX3 suppresses transcription from HBV promoter (Ko et al., 2014). The helicase activity is dispensable for this DDX3-mediated transcription suppression. Thus, DDX3 is identified as a new host restriction factor for HBV.

## ROLE OF DDX3 IN ANTI-VIRAL INNATE IMMUNITY

Viral infection triggers host innate immune responses through activation of the transcription factors NF-κB and IFN regulatory factor (IRF)-3 leading to type I IFN production and anti-viral state in mammalian cells (Gale and Foy, 2005; Saito and Gale, 2007). Similar to NF-κB, IRF-3 is retained in cytoplasm in uninfected cells. After viral infection, IRF-3 is phosphorylated by IKKε and TBK1 and the phosphorylated IRF-3 then homodimerizes and translocates into the nucleus to activate type I IFN. Type I IFNs, such as IFN-α and IFN-β are essential for immune defense against viruses. These IFNs activate the JAK-STAT pathway to induce the IFN-stimulated genes (ISGs), which impact immune enhancing and antiviral action of host cells.

Double-stranded RNA (dsRNA) produced during viral replication is recognized by the host cell as pathogen-associated molecular patterns (PAMPs) by two major pathogen recognition receptor (PRR) proteins: the Toll-like receptors (TLRs; Akira and Takeda, 2004) and DEAD-box RNA helicases RIG-I and Mda5 (Andrejeva et al., 2004; Yoneyama et al., 2004). RIG-I contains two N-terminal caspase activation and recruitment domains (CARD) and a C-terminal RNA helicase domain that binds to dsRNA. Binding viral RNA to RIG-I lead to a conformational change that allows to interact with the RIG-I/Mda5 adaptor IPS-1/MAVS/Cardif/VISA (Kawai et al., 2005; Meylan et al., 2005; Seth et al., 2005; Xu et al., 2005) leading to the activation of IRF-3 and NF-κB. Notably, RIG-I and Mda5 distinguish RNA viruses and are critical for host antiviral responses (Kato et al., 2006). RIG-I is essential for the production of IFN in response to RNA viruses including paramyxoviruses, influenza virus and Japanese encephalitis virus, while Mda5 is critical for picornavirus detection.

**FIGURE 4:**
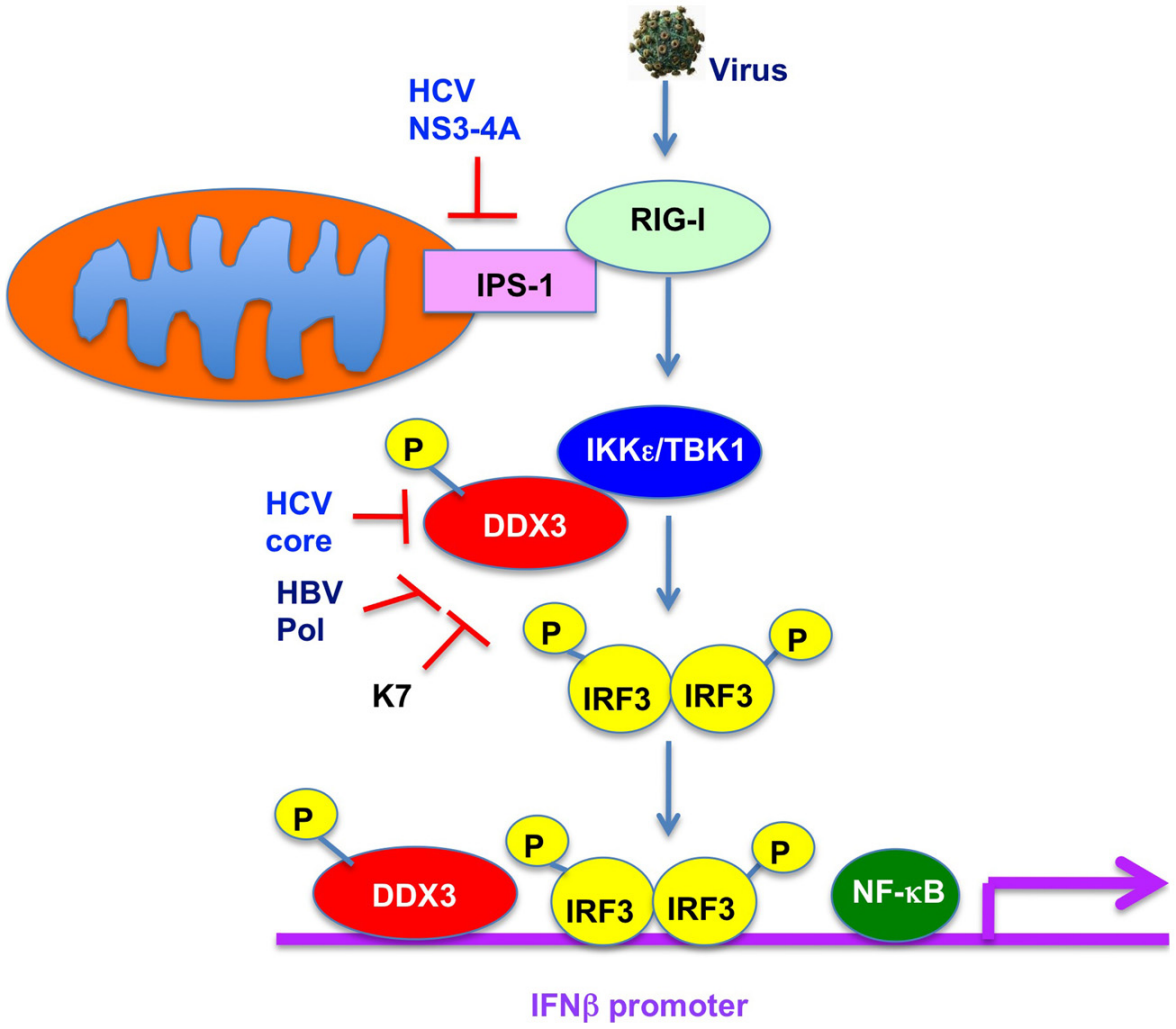
**Role of DDX3 in anti-viral innate immunity.** DDX3 interacts with TBK1/IKKε and is phosphorylated by TBK1/IKKε. TBK1/IKKε then phosphorylates IRF3 and translocates into the nucleus leading to the activation of IFNβ promoter. DDX3 is also recruited on the IFNβ promoter and enhances the IFNβ production. In contrast, HCV core, HBV Pol, or vaccinia virus K7 interacts with DDX3 and suppresses the IFNβ induction.

DDX3 was recently reported to be a component of anti-viral innate immune signaling pathway leading to type I IFN (**Figure 4**; Schröder et al., 2008; Soulat et al., 2008; Gu et al., 2013). Indeed, DDX3 contributes to enhance the induction of anti-viral mediators, IRF3 and type I IFN. DDX3 up-regulates the IFN-β induction through an interaction with IKKε (**Figure 4**; Schröder et al., 2008; Gu et al., 2013) or TBK1 (Soulat et al., 2008). Phosphorylation of DDX3 at serine 102 by IKKε was required for the recruitment of IRF-3 into the complex. Both IKKε and TBK1 are IRF-3-activating kinase to leading the NF-κB and IFN induction. Furthermore, DDX3 is recruited to the IFNβ promoter (**Figure 4**; Soulat et al., 2008), suggesting that DDX3 acts as a transcriptional regulator. In addition, DDX3 also forms a complex with RIG-I and Mda5 and binds to IPS-1 to facilitate IFNβ induction (Oshiumi et al., 2010b), suggesting that DDX3 acts as a viral RNA sensor and a scaffolding adaptor to link of viral RNA with the IPS-1 complex.

In contrast, viruses must overcome the host anti-viral innate immunity. HCV NS3-4A protease cleaves IPS-1/Cardif to block IFNβ induction (**Figure 4**; Meylan et al., 2005) In addition, HCV core protein can disrupt the DDX3-IPS-1/MAVS/Cardif/VISA interaction and act as a viral immune evasion protein preventing IFNβ induction (**Figure 4**; Oshiumi et al., 2010a). Furthermore, DDX3 is known to bind to HBV Pol and restrict the HBV replication (Wang et al., 2009). Conversely, HBV Pol acts as a viral immune evasion protein by disrupting the interaction of DDX3 with TBK1/ IKKε (**Figure 4**; Wang and Ryu, 2010; Yu et al., 2010). Similarly, vaccinia virus K7 protein targets DDX3 (Schröder et al., 2008; Kalverda et al., 2009; Oda et al., 2009) and inhibits the IFNβ induction by preventing TBK1/ IKKε-mediated IRF activation (**Figure 4**; Schröder et al., 2008). Moreover, DDX3 contributes the DNA sensor ZBP1/DAI-dependent IFN response after human cytomegalovirus infection (DeFilippis et al., 2010).

In conclusion, DDX3 participates in anti-viral innate immune signaling pathway leading to type I IFN induction. In contrast, viruses must target DDX3 and evolve mechanisms to overcome this host immune system. Indeed, several RNA viruses sequester and utilize DDX3 for their viral replication and prevent IFN induction.

## Conflict of Interest Statement

The author declares that the research was conducted in the absence of any commercial or financial relationships that could be construed as a potential conflict of interest.
